# Supplementary figures and images for: Molecular mechanism of complement inhibition by the trypanosome receptor ISG65
Source: eLife. 2024 Apr 24;12:RP88960. doi: 10.7554/eLife.88960 (PMC11042801; doi:10.7554/eLife.88960)

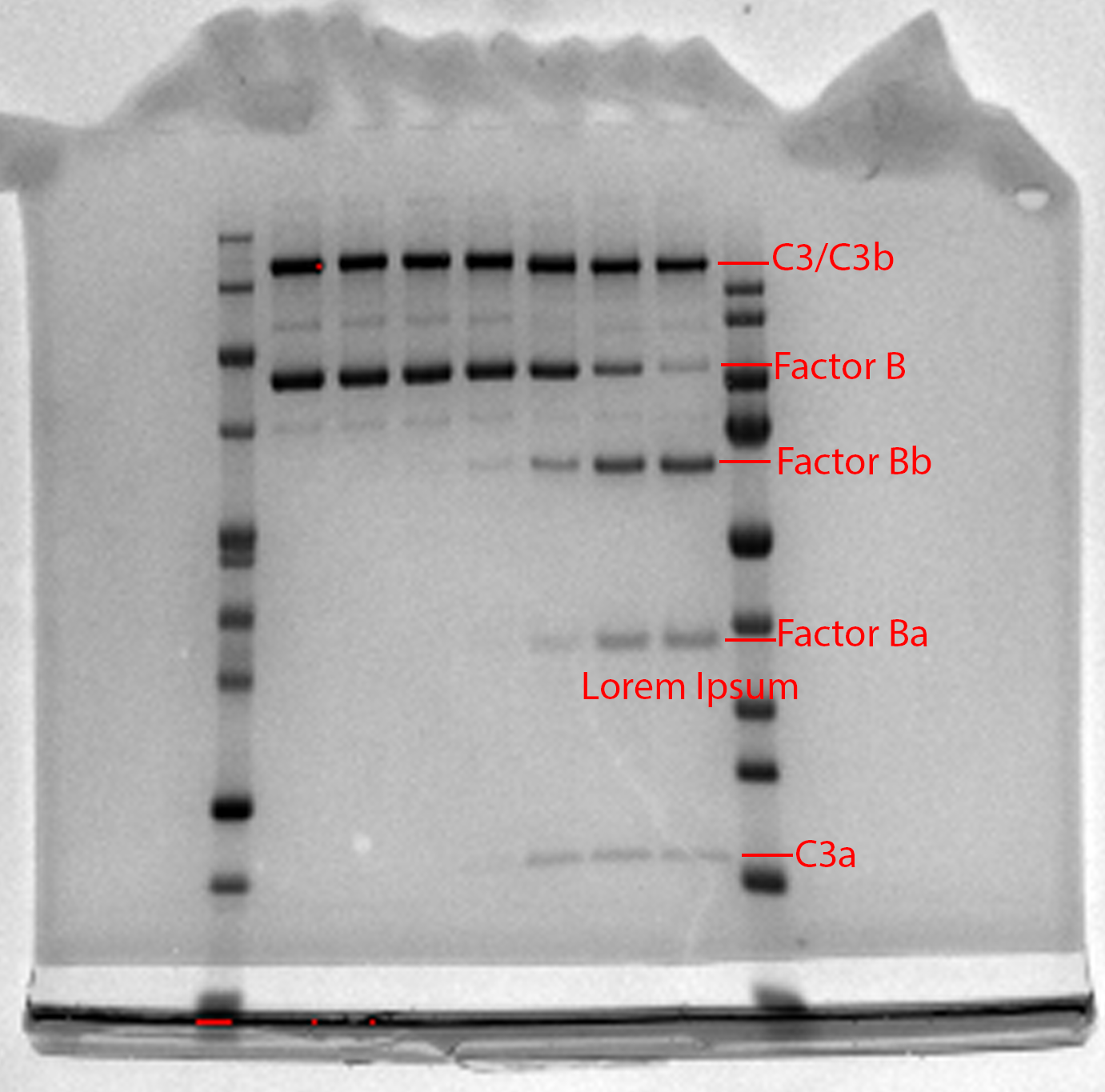

Supplement: Figure 3—source data 1. [file elife-88960-fig3-data1.zip › Figure 3 - source data 1.tif]

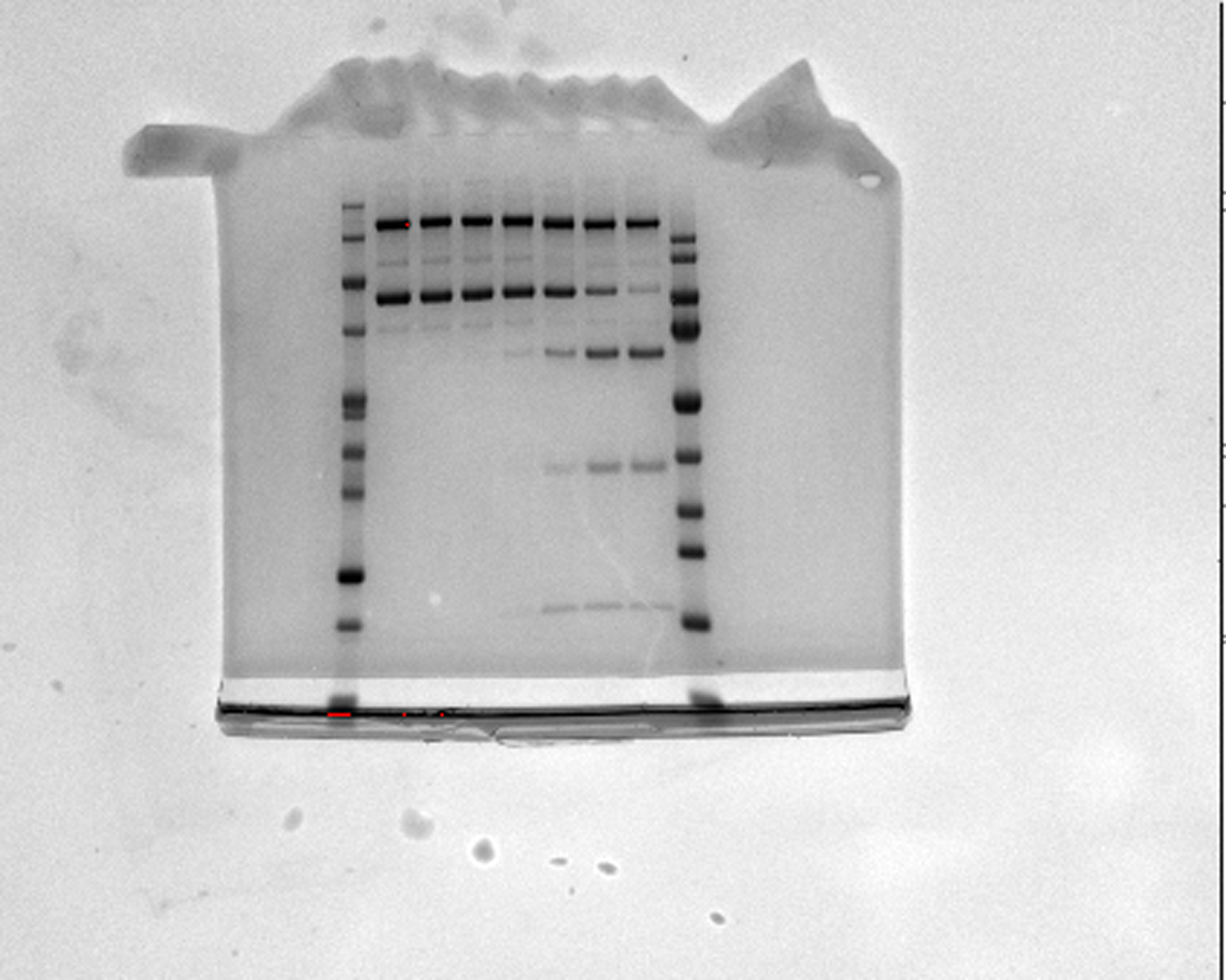

Supplement: Figure 3—source data 2. [file elife-88960-fig3-data2.zip › Figure 3 - source data 2.tif]

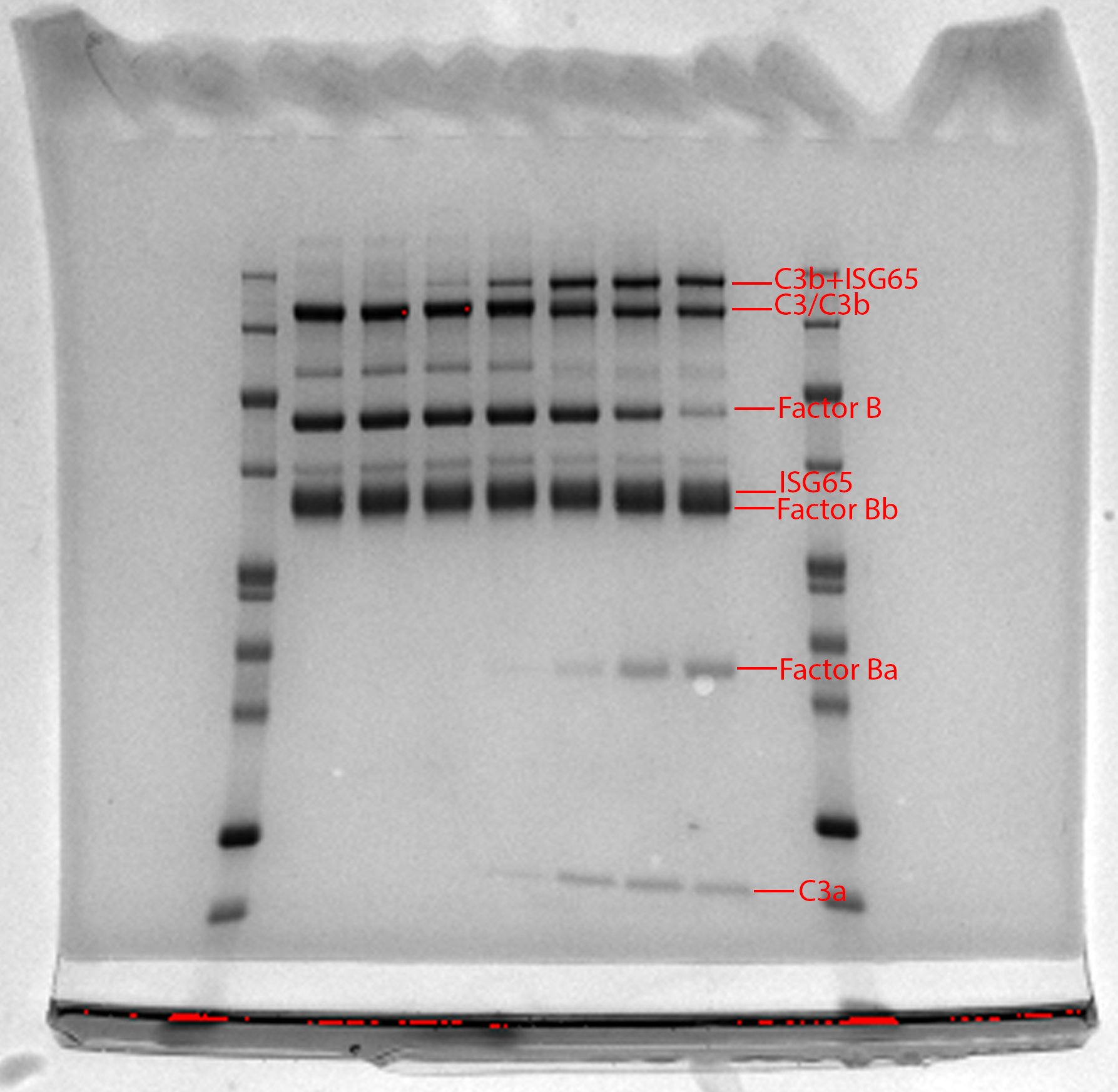

Supplement: Figure 3—source data 3. [file elife-88960-fig3-data3.zip › Figure 3 - source data 3.tif]

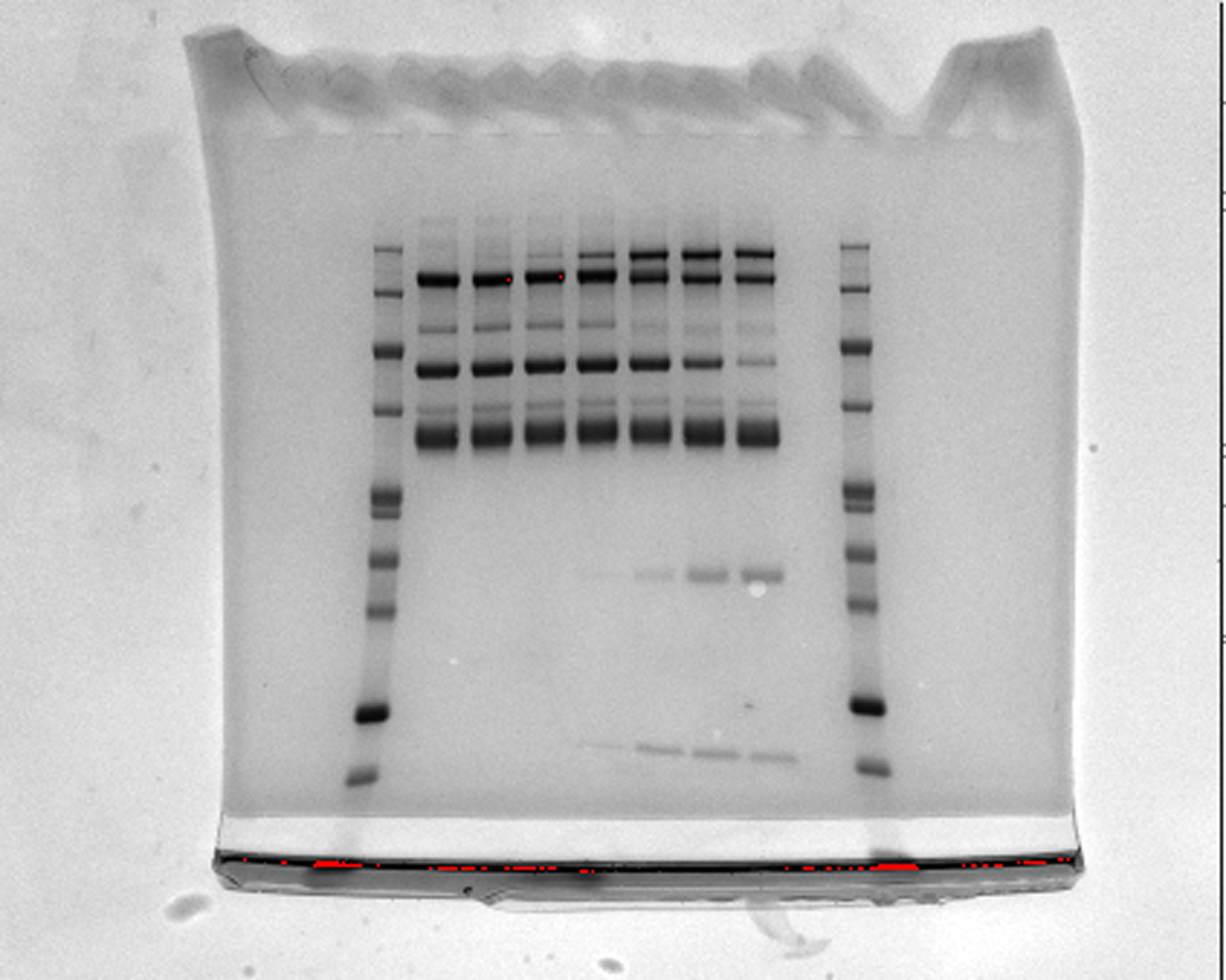

Supplement: Figure 3—source data 4. [file elife-88960-fig3-data4.zip › Figure 3 - source data 4.tif]

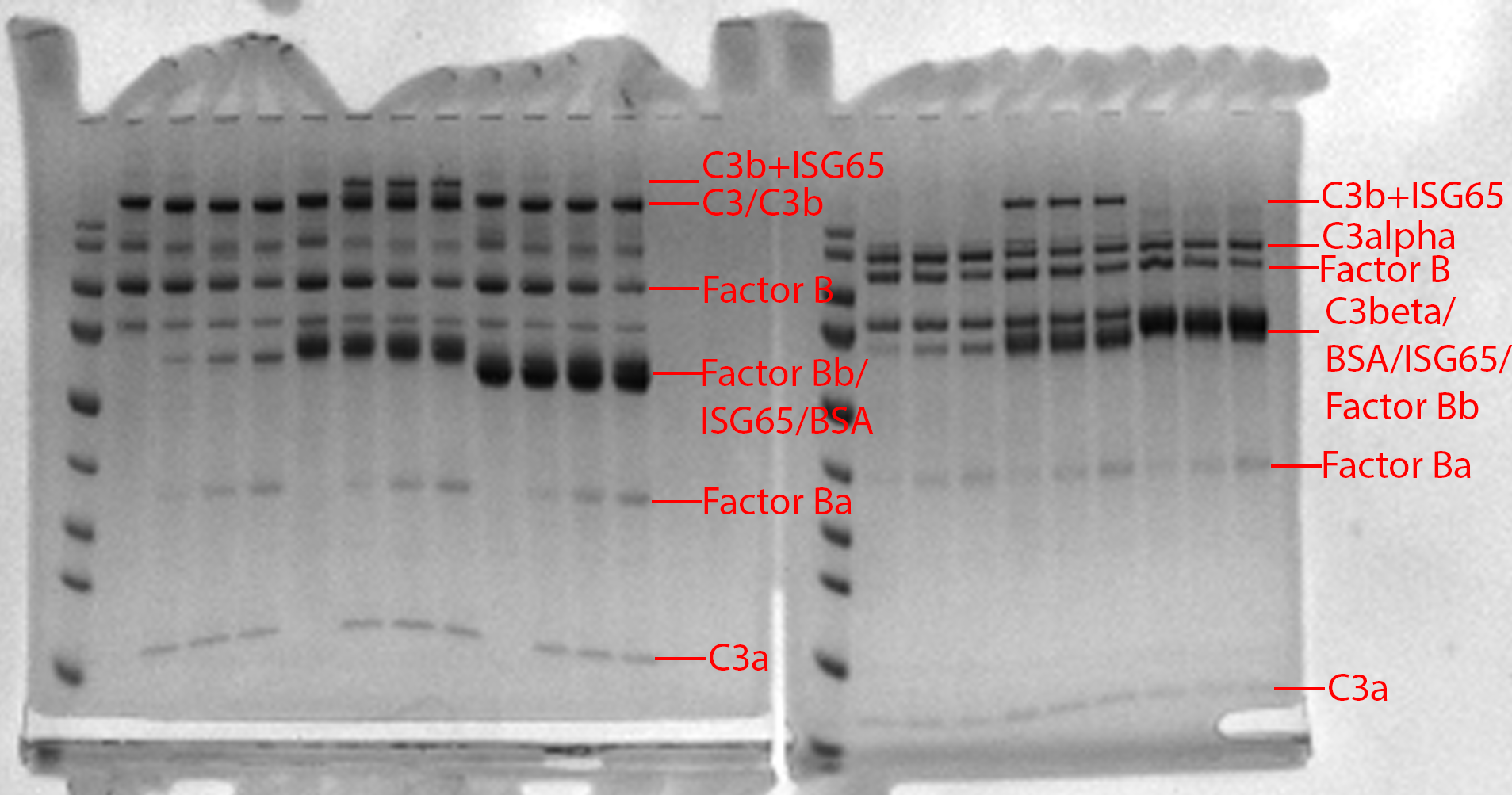

Supplement: Figure 3—source data 5. [file elife-88960-fig3-data5.zip › Figure 3 - source data 5.tif]

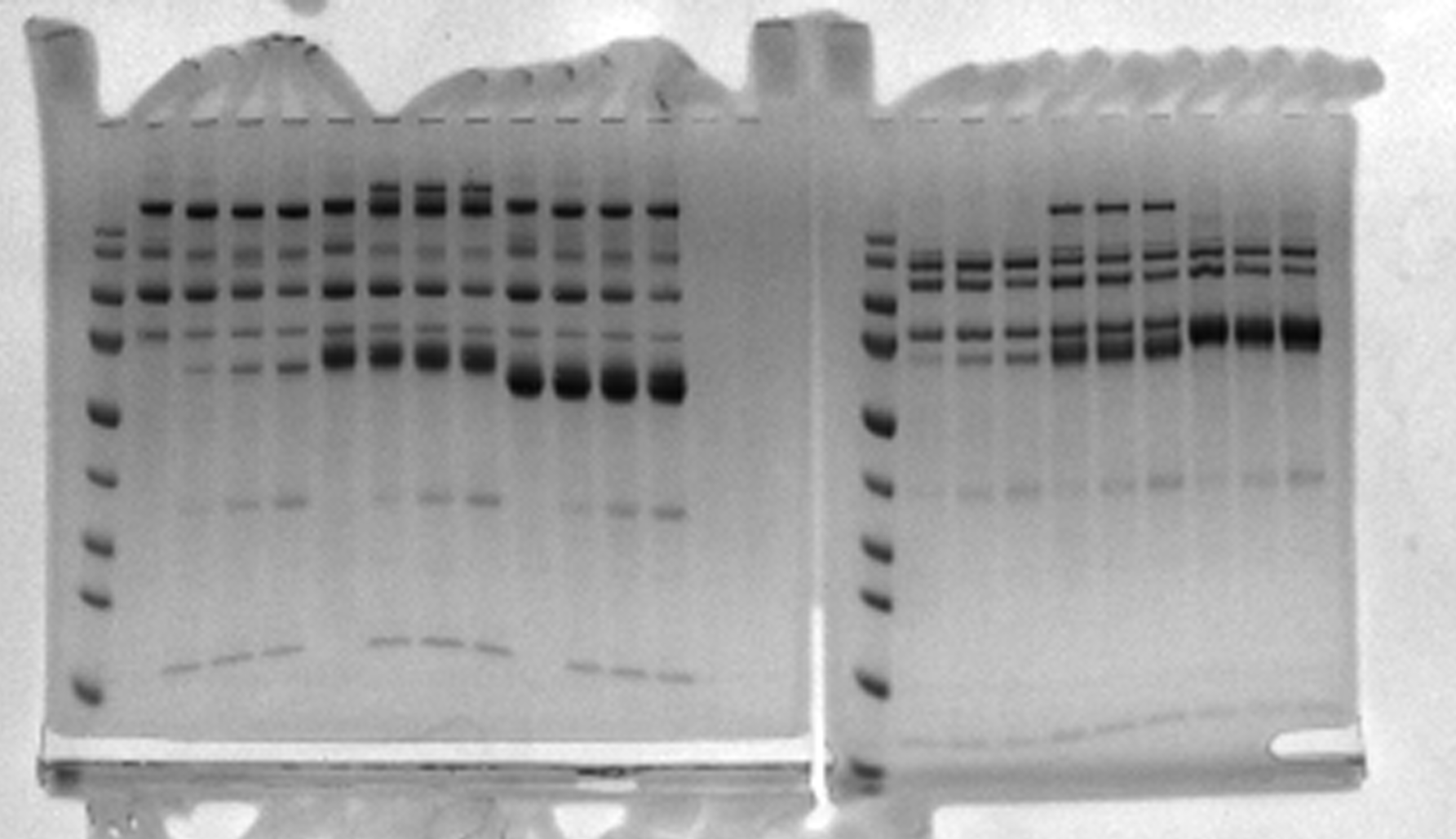

Supplement: Figure 3—source data 6. [file elife-88960-fig3-data6.zip › Figure 3 - source data 6.tif]

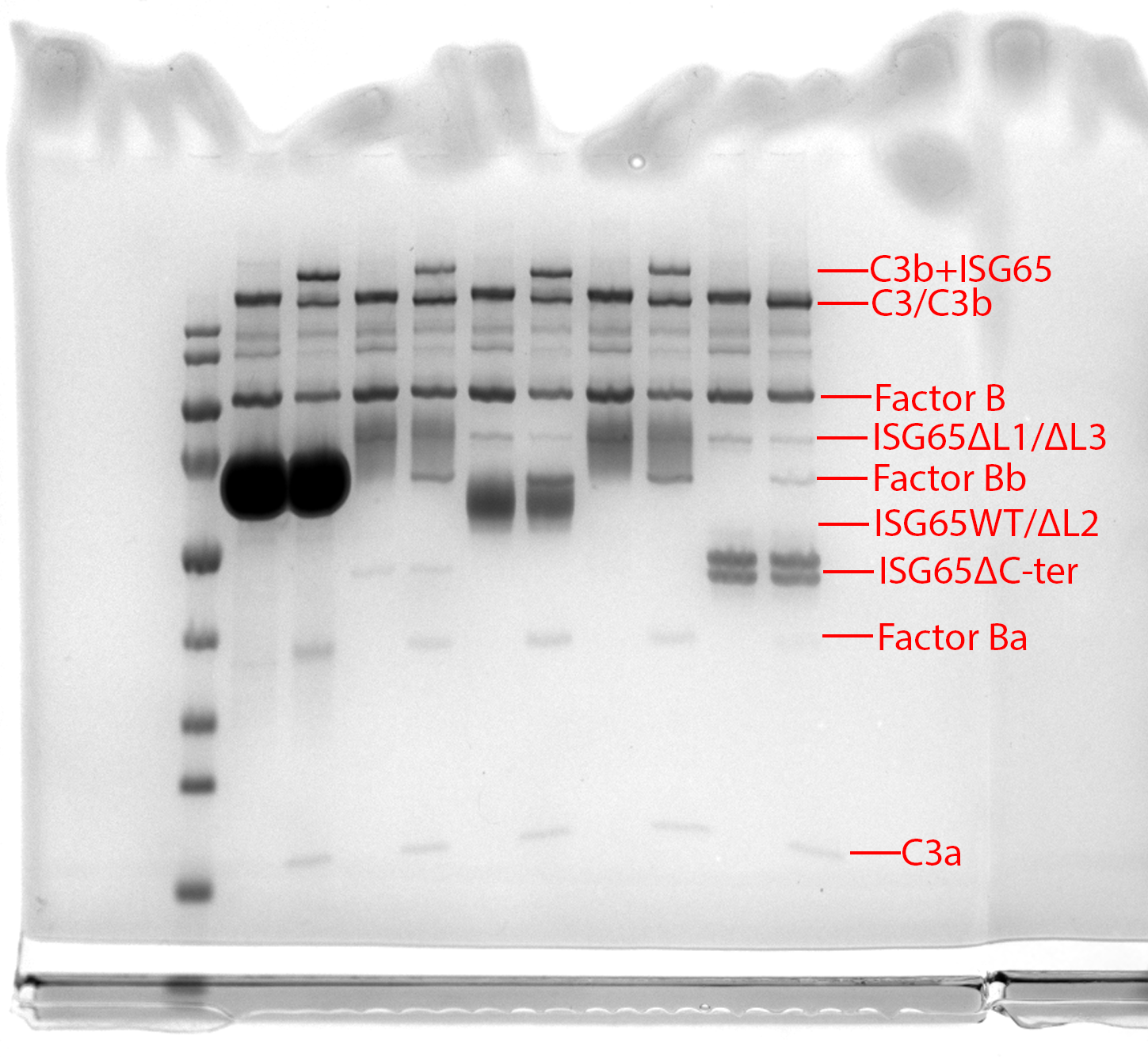

Supplement: Figure 3—source data 7. [file elife-88960-fig3-data7.zip › Figure 3 - source data 7.tif]

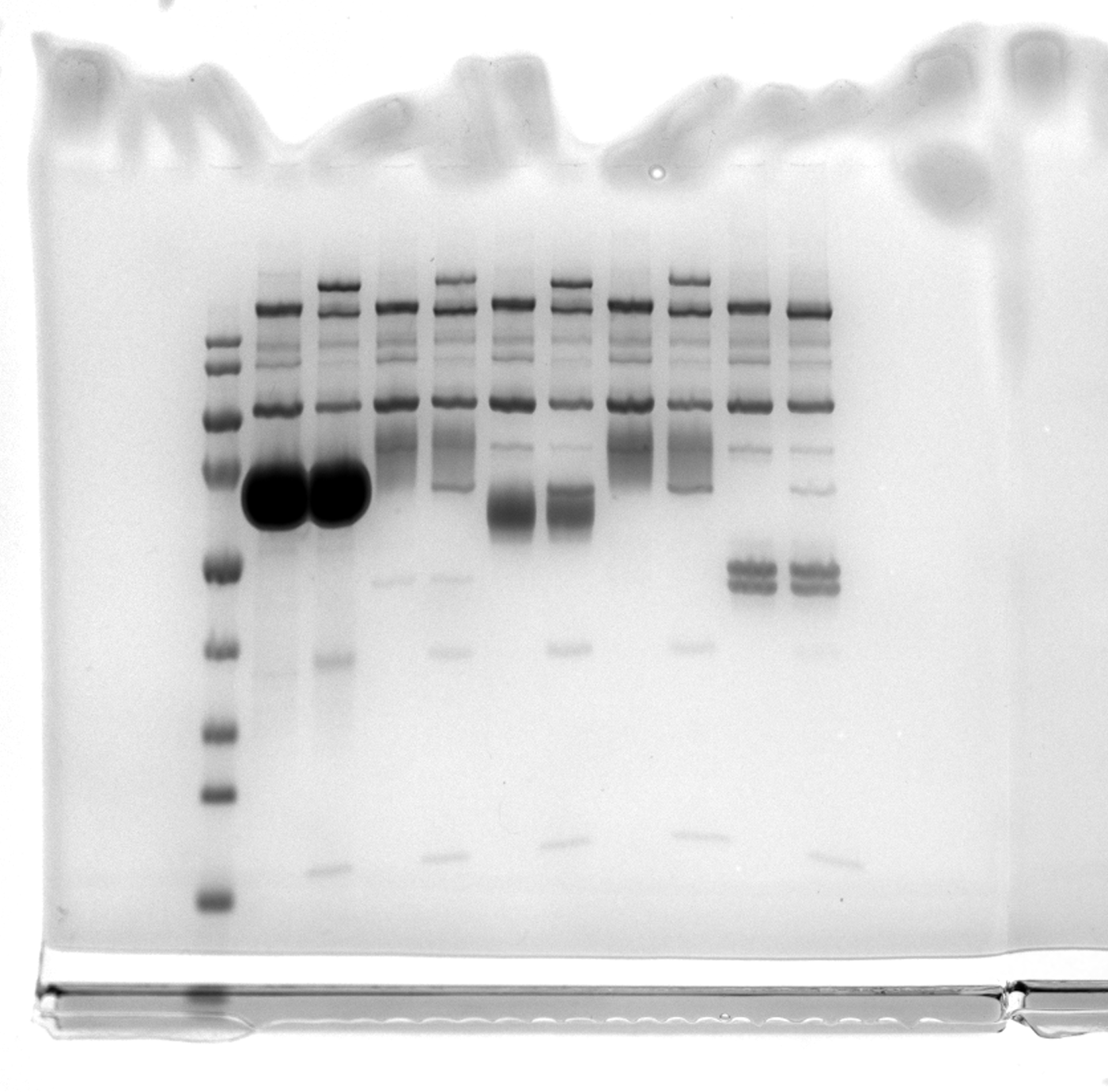

Supplement: Figure 3—source data 8. [file elife-88960-fig3-data8.zip › Figure 3 - source data 8.tif]
