# Supplementary material for: Molecular mechanism of complement inhibition by the trypanosome receptor ISG65
Source: eLife. 2024 Apr 24;12:RP88960. doi: 10.7554/eLife.88960 (PMC11042801; doi:10.7554/eLife.88960)
Supplement: Supplementary file 1. [file elife-88960-supp1.docx]

|  | EMDB-17209 | EMDB-17219 | EMDB-17220 | | EMDB-17221 |  |
| --- | --- | --- | --- | --- | --- | --- |
|  | PDB-8OVB |  |  | |  |  |
|  | **Composite map** | **Local CUB-TED-ISG65**  **refinement** | **Local C3c refinement** | | **C3b only** |  |
| **Data collection** |  |  |  |  | |  |
| Microscope | Titan Krios | | | | |  |
| Detector | Gatan K3 with 20 eV Energy Filter | | | | |  |
| Voltage (kV) | 300 | | | | |  |
| Pixel size (Å) | 0.832 | | | | |  |
| Dose rate (e^–^/Å^2^) | 49 | | | | |  |
| Total exposure (s) | 3.03 | | | | |  |
| Frames per movie | 40 | | | | |  |
| Defocus range (μm) | -1.0 to -3.0 | | | | |  |
|  |  |  |  |  | |  |
| **EM data processing** |  |  |  | |  |  |
| Number of micrographs |  | 14,339 | 14,339 | | 14,339 |  |
| Box size (pixels) |  | 336 | 336 | | 336 |  |
| Initial particle number |  | 3,824,878 | 3,824,878 | | 3,824,878 |  |
| Cleaned particle number |  | 835,488 | 835,488 | | 835,488 |  |
| Final particle number |  | 481,606 | 481,606 | | 382,161 |  |
| Symmetry |  | C1 | C1 | | C1 |  |
| Map resolution (Å) |  | 3.4 | 3.2 | | 3.4 |  |
| FSC threshold |  | 0.143 | 0.143 | | 0.143 |  |
| Map resolution range (Å) |  | 2.9-8.1 | 2.8-7.2 | | 2.8-13.6 |  |
| FSC threshold |  | 0.5 | 0.5 | | 0.5 |  |
| Map postprocessing |  | DeepEMhancer | DeepEMhancer | | DeepEMhancer |  |
| Map combination method | ChimeraX |  |  | |  |  |
|  |  |  |  | |  |  |
| **Model refinement** |  |  |  |  | |  |
| Initial model used | AlphaFold2 (ISG65), 5FO7 (C3b) | | | | | |
| *Model composition* _a_ |  |  |  |  | |  |
| Chains | 3 |  |  |  | |  |
| Protein residues | 1655 |  |  |  | |  |
| Non-hydrogen protein atoms | 13048 |  |  |  | |  |
| *Root mean square Z-score*^a^ |  |  |  |  | |  |
| Bond lengths | 0.29 |  |  |  | |  |
| Bond angles | 0.48 |  |  |  | |  |
| *Validation*^b^ |  |  |  |  | |  |
| Molprobity score | 1.09 |  |  |  | |  |
| Clash score | 2.99 |  |  |  | |  |
| Rotamers favoured (%) | 95.3 |  |  |  | |  |
| Poor outliers (%) | 0.07 |  |  |  | |  |
| *Model vs. Map*^c^ |  |  |  |  | |  |
| FSC (0.5) (Å) | 3.5 |  |  |  | |  |
| CC (mask) | 0.85 |  |  |  | |  |
| *Ramachandran plot*^b^ |  |  |  |  | |  |
| Favoured (%) | 98.1 |  |  |  | |  |
| Outliers (%) | 0 |  |  |  | |  |

^a^ Statistics calculated using the PDB validation server

^b^ Statistics calculated using Molprobity^50^

^c^ Statistics calculated using PHENIX^51^
