# Supplementary material for: Molecular mechanism of complement inhibition by the trypanosome receptor ISG65
Source: eLife. 2024 Apr 24;12:RP88960. doi: 10.7554/eLife.88960 (PMC11042801; doi:10.7554/eLife.88960)
Supplement: Supplementary file 2. — Average values and standard deviation from three experimental repeats are reported. [file elife-88960-supp2.docx]

| Immobilised biotin-C3b | | | |
| --- | --- | --- | --- |
|  | ISG65 | ISG65∆L2 | ISG65^N188A,H189A,Y190A^ |
| K_D_ (μM) | 2.9 ± 1.5 | 8.1 ± 4 | 7.8 ± 0.7 |
| kon_1_ (M^-1^s^-1^) | 23085 ± 8977 | 21469 ± 7843 | 20942 ± 5356 |
| koff_1_ (s^-1^) | 0.06 ± 0.02 | 0.16 ± 0.04 | 0.16 ± 0.05 |
| Chi^2^ (RU^2^) | 11.4 ± 6 | 8.8 ± 5 | 3.8 ± 4.1 |
| Immobilised biotin-C3d | | | |
|  | ISG65 | ISG65∆L2 | ISG65^N188A,H189A,Y190A^ |
| K_D_ (μM) | 6 ± 2.6 | 6.5 ± 1.2 | 6.7 ± 0.6 |
| kon_1_ (M^-1^s^-1^) | 16543 ± 5465 | 22462 ± 5908 | 23079 ± 6569 |
| koff_1_ (s^-1^) | 0.09 ± 0.04 | 0.14 ± 0.02 | 0.15 ± 0.04 |
| Chi^2^ (RU^2^) | 18.8 ± 17.2 | 14.9 ± 3.6 | 2.5 ± 1.2 |
