# Supplementary material for: Molecular mechanism of complement inhibition by the trypanosome receptor ISG65
Source: eLife. 2024 Apr 24;12:RP88960. doi: 10.7554/eLife.88960 (PMC11042801; doi:10.7554/eLife.88960)
Supplement: Supplementary file 3. — The high molecular weight band observed in convertase assay in the presence of ISG65 was excised and analysed by mass spectrometry. The three most abundant proteins identified are presented. [file elife-88960-supp3.docx]

| **Description** | **Uniprot Accession** | **Score** | **Coverage (%)** |
| --- | --- | --- | --- |
| Complement C3 | P01024 | 6495 | 32 |
| ISG65 | A0A8J9S0Z8 | 2601 | 31 |
| Factor B | P00751 | 160 | 4 |
