# Supplementary material for: Molecular mechanism of complement inhibition by the trypanosome receptor ISG65
Source: eLife. 2024 Apr 24;12:RP88960. doi: 10.7554/eLife.88960 (PMC11042801; doi:10.7554/eLife.88960)
Supplement: Supplementary file 4. [file elife-88960-supp4.docx]

| **Factor B primers for Gibson Assembly into pHL-SEC, and adding a C-tag.** | |
| --- | --- |
| Forward (5’ to 3’) | ttgcgtagctactccatggtctttggcc |
| Reverse (5’ to 3’) | GTGATCATTAAGCTTCTGGTTCGCCAGAGCCTAGAAAACCCAAATCCTCATCTTG |
| **pHL-SEC primers for Gibson Assembly of Factor B pHL-SEC, and adding a C-tag.** | |
| Forward (5’ to 3’) | accagaagcttaatgatcactcgagactagtatc |
| Reverse (5’ to 3’) | accatggagtagctacgcaacccatcag |
| **Factor D primers for Gibson Assembly into pHL-SEC, and adding a C-tag.** | |
| Forward (5’ to 3’) | gatgggttgcgtagctgctcctcctagaggaagaatc |
| Reverse (5’ to 3’) | GATCATTAAGCTTCTGGTTCGCCAGAGCCGGCCAGCACAGAATCGATCCAG |
| **pHL-SEC primers for Gibson Assembly of Factor D pHL-SEC, and adding a C-tag.** | |
| Forward (5’ to 3’) | cagaagcttaatgatcactcgagactagtatc |
| Reverse (5’ to 3’) | agctacgcaacccatcag |
| **ISG65 primers for introducing ΔL1 deletion and adding a serine linker by Gibson Assembly** | |
| Forward (5’ to 3’) | CGGCTCTTCAGGCTATGCCAAGCTGAGCG |
| Reverse (5’ to 3’) | CCTGAAGAGCCGTTCAGCTTTTCCAGCC |
| **ISG65 primers for introducing ΔL2 deletion by Gibson Assembly** | |
| Forward (5’ to 3’) | GACCACAAGAGCGCCATCGACTGTAGC |
| Reverse (5’ to 3’) | CGATGGCGCTCTTGTGGTCATCCAGGTCCTGG |
| **ISG65 primers for introducing ΔL3 deletion by Gibson Assembly** | |
| Forward (5’ to 3’) | GAAGCCCAAGTGCACCATGACAGAGGAATGGC |
| Reverse (5’ to 3’) | TCATGGTGCACTTGGGCTTCACGTTCTCCCAG |
| **ISG65 primers for introducing ΔC-ter deletion by Gibson Assembly** | |
| Forward (5’ to 3’) | agaggccggaggactgaacgacatcttcgag |
| Reverse (5’ to 3’) | cgttcagtcctccggcctctttagcagc |
| **ISG65 primers for introducing ISG65^N188A,H189A,Y190A^ mutation by Gibson Assembly** | |
| Forward (5’ to 3’) | GCAGCCCACGCCCTGAGCGTGAACAGAAGCGCCATCG |
| Reverse (5’ to 3’) | CAGGGCGTGGGCTGCGAACTGGATGCCGTCGCAGTTAGG |
| **C3d primers for introducing C3d^A1010C,Q1013A^ mutation by Gibson Assembly** | |
| Forward (5’ to 3’) | TTGCGGTGAAGCGAATATGATTGGTATGACCCCGACCGTTATTG |
| Reverse (5’ to 3’) | CATATTCGCTTCACCGCAACCGCTCGGGGTAACAATCAGATG |
